# Plunging Ranula in a 78- year- old Male – a Rare Case Report 

**DOI:** 10.4317/jced.54114

**Published:** 2018-01-01

**Authors:** Arunkumar Kamalakaran, Balaji Jayaraman, Saravanan Balasubramaniam, Rohini Thirunavukkarasu, Bharathi Ramakrishnan

**Affiliations:** 1 Associate Professor, Department of Oral and Maxillofacial surgery, Tamilnadu government Dental College and Hospital, Chennai, India; 2 Professor, Department of oral and maxillofacial Surgery, Tamilnadu Government Dental College and Hospital, Chennai, India; 3 Principal,Tamilnadu government Dental College and Hospital, Chennai, India; 4 Assistant Professor, Department of oral and maxillofacial Surgery, Tamilnadu Government Dental College and Hospital, Chennai, India; 5 Professor, Department of Oral Pathology and Microbiology, Tamilnadu government Dental College and Hospital, Chennai, India

## Abstract

The term Ranula is a Latin word meaning frog. It refers to a bluish translucent cystic lesion in the floor of the mouth resembling the underbelly of a frog. Ranulas can be true cysts occurring due to ductal obstruction of the sublingual gland or a minor salivary gland or a pseudocyst as a result of ductal injury leading to extravasation and accumulation of saliva in the surrounding tissues. Clinically ranulas present as intraoral or plunging ranulas. The prevalence of ranula is 0.2% per 1000 patients Ranulas account for 6% of all salivary gland cysts. Ranulas are more common in children and young adults. However the plunging type occurs most commonly in the later third decade. The diagnosis of plunging ranula is based on a combined clinical,radiographic imaging and histologic findings. The treatment of ranulas have always been controversial.The treatment modalities range from simple marsupialisation to excision of the pseudocyst along with sublingual or submandibular gland excision.The purpose of this paper is to present a rare case of plunging ranula and to highlight the importance of considering plunging ranula in the differential diagnosis of lesions of the neck.

** Key words:**Plunging Ranula,mucus, submandibular gland, transcervical approach.

## Introduction

The term ranula is a Latin word meaning frog (1). It refers to a bluish translucent cystic lesion in the floor of the mouth resembling the underbelly of a frog. Ranulas can be true cysts occurring due to ductal obstruction of the sublingual gland or a minor salivary gland or a pseudocyst as a result of ductal injury leading to extravasation and accumulation of saliva in the surrounding tissues (2). Clinically ranulas present as intraoral or plunging ranulas. Plunging ranula occurs when the fluid pressure of the mucus dissects through a perforation in the mylohyoid muscle to the submandibular space. The diagnosis of plunging ranula is based on a combined clinical,radiographic imaging and histologic findings. The treatment of ranulas have always been controversial.The treatment modalities range from simple marsupialisation to excision of the pseudocyst along with sublingual or submandibular gland excision (3).

The purpose of this paper is to present a rare case of plunging ranula and to highlight the importance of considering plunging ranula in the differential diagnosis of lesions of the neck.

## Case Report

**Figure 1:**
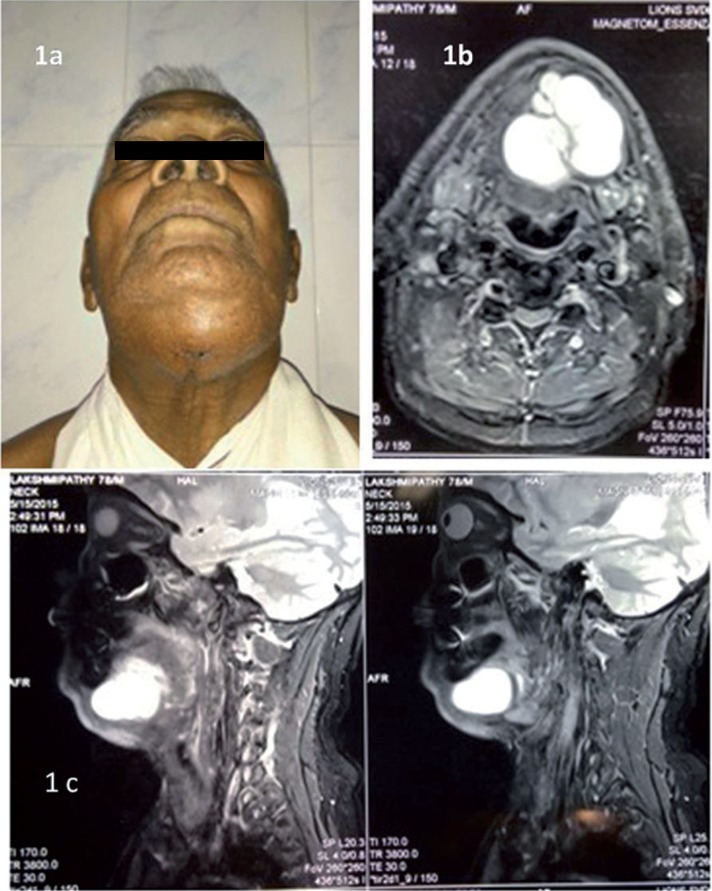
a) Extraoral view showing swelling in the submental region extending bilaterally to the submandibular region. b,c) Magnetic resonance imaging axial and sagittal view showing well defined altered signal intensity soft tissue mass lesion involving the floor of the mouth with extension into the submental region displacing the left submandibular gland.

**Figure 2:**
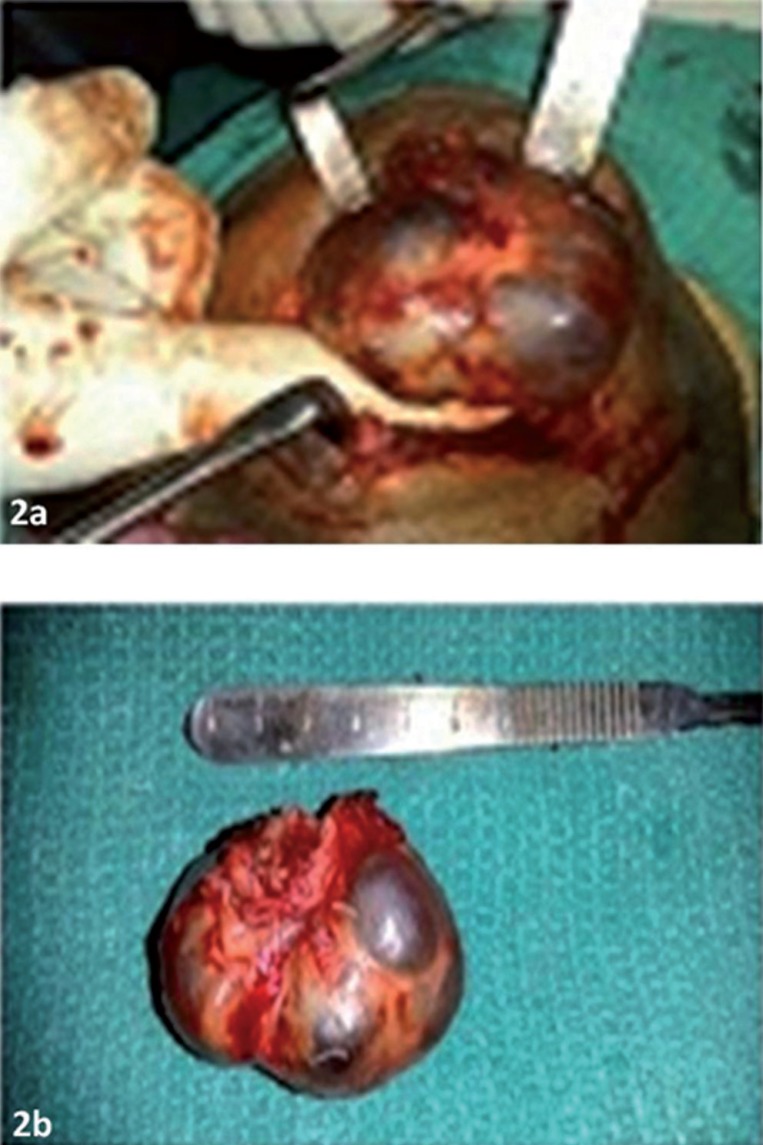
a,b) Excised lesion in toto.

**Figure 3:**
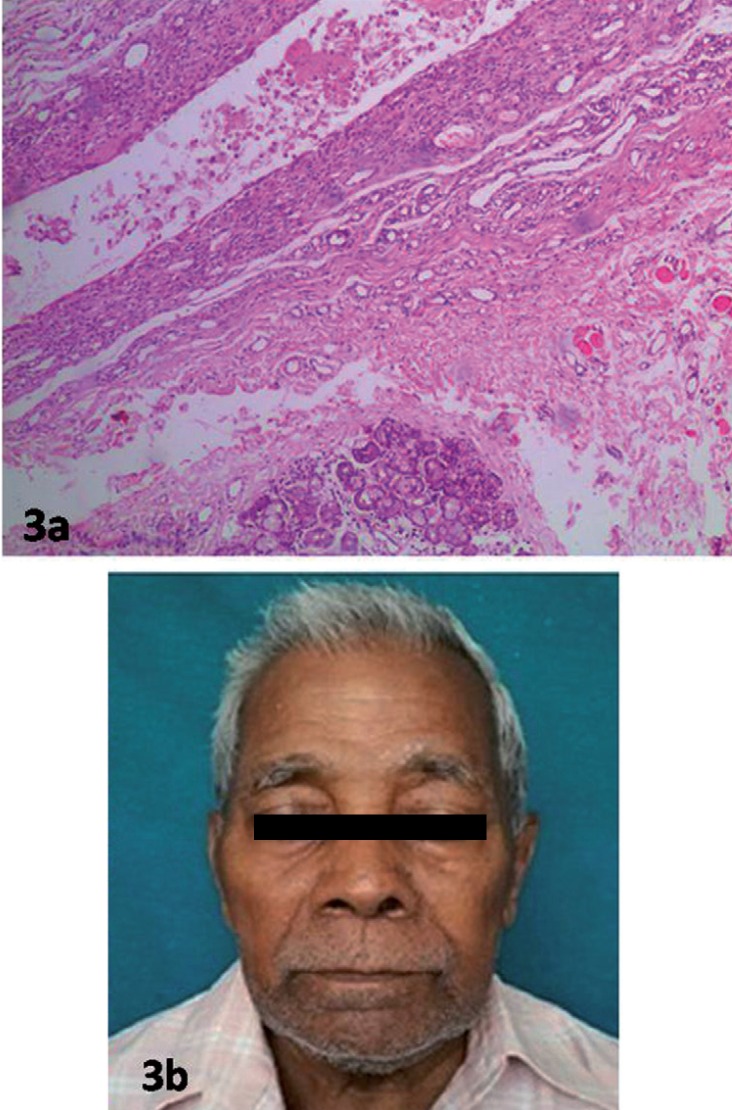
a) Histopathology of Plunging Ranula (H and E)-Pseudocystic cavity filled with mucin and mucinophages surrounded by capsule and adjacent minor salivary gland tissue-(×100). b) Postoperative extraoral view showing no evidence of relapse after two year follow up.

A 78- year- old male was referred with the chief complaint of a painless swelling in the lower jaw since 2 months. Extraoral examination revealed a swelling of size 5×4 cm in the submental region extending posteriorly bilaterally till the submandibular region (Fig. 1a). The swelling was firm in consistency with an intact overlying skin. Intraorally no visible swelling was evident however on palpation there was mucous discharge from the left Wharton’s duct. A provisional diagnosis of salivary gland neoplasm was made. The differential diagnosis included plunging ranula and sublingual dermoid cyst. An MRI was advised and the report suggested a well defined altered signal intensity soft tissue mass lesion involving the floor of the mouth with extension into the submental region displacing the left submandibular gland (Fig. 2a,b). An incisional biopsy was done and histopathology report was suggestive of a ranula. Taking into consideration the clinical presentation, the MRI and the histopathology report, a final diagnosis of plunging ranula was made. Excision of the lesion under general anesthesia was planned. A cervical incision was made, layerwise dissection was done, lesion was identified and excised in toto after releasing the adhesions (Fig. 2a,b). The specimen was subjected to post operative histopathological examination. The post-operative report was consistent with the preoperative report confirming the diagnosis of ranula showing pseudocystic cavity filled with foamy histiocytes, extravasated RBC’s (Fig. 3). The patient is disease free after a two- year follow -up.

## Discussion

A Ranula is a mucus- filled cavity arising in relation to the sublingual gland. Ranulas are classified based on their clinical presentation as sublingual,plunging and sublingual-plunging ranulas (4). Sublingual ranulas present as bluish translucent swellings in the floor of the mouth .In contrast plunging ranulas present cervically because of their extension beyond the mylohyoid muscle and sublingual –plunging type present both intraorally and cervically (5). In the current case since the lesion predominantly had a cervical presentation with no typical intraoral translucent swelling, it was classified as plunging type. The prevalence of ranula is 0.2% per 1000 patients (6). Ranulas account for 6% of all salivary gland cysts (6). Ranulas are more common in children and young adults. However, the plunging type occurs most commonly in the later third decade (6) but a presentation at an age of 78 as in the current case is very rare.

The plunging ranulas may occur as a result of the projection of the sublingual gland through the mylohyoid or from an ectopic salivary gland existing on the cervical side of the mylohyoid (7). This is considered to be the cause for plunging ranulas without an oral component . In the present case this could be explained as the cause since there was no intra oral presentation or the cyst may penetrate through the dehiscence in the mylohyoid which may be iatrogenically induced or a duct from the sublingual gland may join the submandibular gland or its duct, allowing ranulas to form in continuity with the submandibular gland (8).

The diagnostic tools used for the diagnosis of plunging ranulas include FNAC, CT scan, and MRI. Takimoto (9) reported a simple radiographic technique for the diagnosis of plunging ranulas by injecting radiopaque contrast medium into sublingual space. Ultrasonography is usually inconclusive because of its location. On CT scan they appear as a rough or ovoid shaped cystic lesion with a homogenous central accentuation (10). However, MRI is suggested to be the most sensitive method for the diagnosis of plunging ranula. The ranulas because of high water content appear hypointense in T1 weighted images and hyperintense in T2 weighted images (11) as in the present case yet the signal intensity may vary depending on the protein concentration in which case a diagnosis of lipoma or dermoid or epidermoid cyst could be made. ‘Tail sign’ which is seen as the result of the tapered continuation of the cervical portion of the ranula into the sublingual space on CT and MRI imaging is said to be pathognomonic of a plunging ranula (11). However, a cystic hygroma invading the sublingual space gives an appearance similar to the ‘tail sign’. Since no ‘tail sign’ was evident in the present case, this sign cannot be considered universal.

Histologically ranula appears as a cyst with no epithelial lining and a vascular fibrous connective tissue wall containing chronic inflammatory cells and macrophages filled with mucin (12). Similar histological findings have been reported in this case. Literature (13) suggests that a biopsy is mandatory for ranulas not only for histopathological diagnosis but also to rule out squamous cell carcinoma arising from the cyst wall or papillary cyst adenocarcinoma of the sublingual gland.

The treatment options for plunging ranula includes sclerotherapy with OK-432, excision of the ranula,sublingual gland and ranula excision or sublingual gland excision (14).

Fukase *et al.* (15)reported 100% resolution of 11 cases of plunging ranula after multiple injections of OK432. On the contrary Rho *et al.* (16) reported a success rate of only 33.3% after one injection of OK432 in their series. Davison et al. (17) and Ichimura *et al.* (18) in their review of five cases each on the treatment of plunging ranula by excision of the ranula alone reported no recurrence but complications such as paraesthesia of the tongue and infection. Plunging ranulas can be approached through a transcervical or transoral approach (19). The complications associated with a cervical approach include injury to the marginal mandibular nerve, cervical fistula, and scarring. With an intraoral approach, the complications encountered are an injury to the lingual nerve,Wharton’s duct, hematoma and bleeding. When excision of the sublingual gland and the ranula is planned a transcervical approach may be difficult because it requires the division of the mylohyoid muscle and dissection up to the mucosa of the floor of the mouth. CO2 lasers have been used to vaporize ranulas (20).

The reported recurrence rates after various treatment modalities are incision and drainage (70% to 100%), marsupialization (36.4% to 80%), excision of ranula only (18.7% to 85%), and excision of ranula along with sublingual salivary gland (0% to 3.8%) (21). In this case taking into consideration, the age of the patient excision of the ranula alone through a cervical approach was performed.

## Conclusions

Plunging ranula is a rare entity which should be considered in the differential diagnosis of neck lesions. A comprehensive diagnosis of plunging ranula can be made only on a combination of clinical , imaging and histologic findings. The treatment of plunging ranula is controversial and the treatment modality with the lowest recurrence rate and minimum morbidity should be planned. The choice of treatment should be decided on an individualistic basis rather than a fixed treatment protocol.
